# 2-[1-({2-[1-(2-Hy­droxy-5-{[meth­yl(phen­yl)amino]­meth­yl}phen­yl)ethyl­idene­amino]­eth­yl}imino)­eth­yl]-4-{[meth­yl(phen­yl)amino]­meth­yl}phenol

**DOI:** 10.1107/S1600536812017904

**Published:** 2012-04-28

**Authors:** Ali Ourari, Yasmina Ouennoughi, Sofiane Bouacida

**Affiliations:** a Laboratoire d’Electrochimie, d’Ingénierie Moléculaire et de Catalyse Redox (LEIMCR), Faculté des Sciences de l’Ingénieur, Université Farhat Abbas, Sétif 19000, Algeria; b Unité de Recherche de Chimie de l’Environnement et Moléculaire Structurale (CHEMS), Université Mentouri-Constantine, 25000 Algeria

## Abstract

Mol­ecules of the title compound, C_34_H_38_N_4_O_2_, lie across crystallographic inversion centres. The crystal packing can be described by alternating zigzag chains along the *c* axis in which the molecules are linked by van der Waals interactions. There is an intra­molecular O—H⋯N hydrogen bond and the two benzene rings in the asymmetric unit make a dihedral angle of 79.81 (6)°.

## Related literature

For the synthesis and applications of similar compounds and derivates containing both an anilinic moiety and a salicyl­aldehyde derivative, see: Wulff & Akelah (1979 ▶); Horwitz & Murray (1988 ▶); Smith *et al.* (2003 ▶); Dong *et al.* (2010 ▶); Guo & Wong (1999 ▶); Stejskal & Gilbert (2002 ▶); Coche-Guerente *et al.* (1996 ▶); Ourari *et al.* (2008 ▶); Khedkar & Radhakrishnan (1997 ▶); Huo *et al.* (1999 ▶).
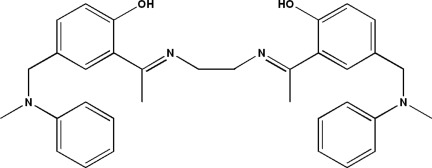


## Experimental

### 

#### Crystal data

C_34_H_38_N_4_O_2_
*M* *_r_* = 534.68
Orthorhombic, 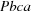

*a* = 7.460 (1) Å
*b* = 12.350 (1) Å
*c* = 31.176 (2) Å
*V* = 2872.3 (5) Å^3^
*Z* = 4
Mo *K*α radiation
μ = 0.08 mm^−1^
*T* = 295 K
0.15 × 0.08 × 0.04 mm

#### Data collection

Nonius KappaCCD diffractometer
5411 measured reflections
2916 independent reflections
1698 reflections with *I* > 2σ(*I*)
*R*_int_ = 0.023

#### Refinement

*R*[*F* ^2^ > 2σ(*F* ^2^)] = 0.063
*wR*(*F* ^2^) = 0.216
*S* = 1.01
2916 reflections
185 parameters
H-atom parameters constrained
Δρ_max_ = 0.30 e Å^−3^
Δρ_min_ = −0.15 e Å^−3^

### 

Data collection: *COLLECT* (Nonius, 1998 ▶); cell refinement: *SCALEPACK* (Otwinowski & Minor, 1997 ▶); data reduction: *DENZO* (Otwinowski & Minor 1997 ▶) and *SCALEPACK*; program(s) used to solve structure: *SIR2002* (Burla *et al.*, 2005 ▶); program(s) used to refine structure: *SHELXL97* (Sheldrick, 2008 ▶); molecular graphics: *ORTEP-3 for Windows* (Farrugia, 1997 ▶) and *DIAMOND* (Brandenburg & Berndt, 2001 ▶); software used to prepare material for publication: *WinGX* (Farrugia, 1999 ▶).

## Supplementary Material

Crystal structure: contains datablock(s) global, I. DOI: 10.1107/S1600536812017904/vm2172sup1.cif

Structure factors: contains datablock(s) I. DOI: 10.1107/S1600536812017904/vm2172Isup2.hkl

Supplementary material file. DOI: 10.1107/S1600536812017904/vm2172Isup3.cml

Additional supplementary materials:  crystallographic information; 3D view; checkCIF report

## Figures and Tables

**Table 1:** Hydrogen-bond geometry (Å, °)

*D*—H⋯*A*	*D*—H	H⋯*A*	*D*⋯*A*	*D*—H⋯*A*
O9—H9⋯N1	0.82	1.79	2.517 (2)	147

**Fig. 1.**
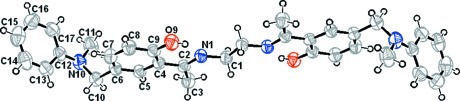
The molecular geometry of (I) with displacement ellipsoids drawn at the 50% probability level. H atoms are represented as small spheres of arbitrary radii. Only the non-H atoms of the asymmetric unit are labelled.

**Fig. 2.**
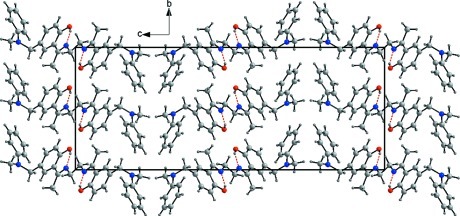
Packing diagram of (I) viewed along the a axis showing alterning chains and intramolecular O—H···N hydrogen bond interaction (shown in red).

## supplementary crystallographic information

### Comment

The synthesis of new derivatives containing simultaneously an anilinic moiety and salicylaldehyde derivative is of great interest given that they are currently used as precursors for the preparation of chelating agents such as Schiff bases (Wulff & Akelah, 1979; Horwitz *et al.*, 1988) and oximes (Smith *et al.*, 2003; Dong *et al.*, 2010). These compounds may also be involved in the elaboration of modified electrodes by anodic (Guo *et al.*, 1999) or by chemical oxidation (Stejskal *et al.*, 2002). These materials are mainly applied in catalysis, electrocatalysis and sensors (Ourari *et al.*, 2008; Coche-Guerente *et al.*, 1996). The synthesis of new salicylaldehyde derivatives containing electropolymerizable units can be considered as the main source of functionalized conducting π-conjugated polymers as those of polyaniline and polypyrrole (Huo *et al.*, 1999; Khedkar *et al.*, 1997).

We report here the synthesis of title compound and its crystal structure. The molecular geometry of (I), and the atomic numbering used, is illustrated in Fig. 1. The asymmetric unit of the title compound, (I), consists of one-half of the molecule, with the other half generated by a crystallographic inversion centre. The two phenyl rings make a dihedral angle of 79.81 (6)°. The crystal packing in the title structure can be described by alterning zigzag chains along the *c* axis (Fig. 2). There is an intramolecular O—H···N hydrogen bonding (Table 1, Fig. 2) and the packing is stabilized by Van der Waals interactions and weak π···π stackings (minimal distance: Cg1··· Cg2^i^= 5.2048 (15) Å; Cg1 and Cg2 are the centroids of rings C4-C9 and C12-C17, respectively; symmetry code: (i) -1 + *x*, *y*, *z*). These interactions link the molecules within the chains and also link the layers together reinforcing the cohesion of the structure.

### Experimental

The title compound is a tetradentate Schiff base ligand (H2*L*). It was synthesized by dissolving 7 g (27.45 mmol) of 5-(*N*,*N*-methylphenylaminomethyl)-2-hydroxyacetophenone in 30 ml of absolute ethanol and placed in a three-necked flask of 100 ml, surmounted by a condenser. To this solution, 0.823 g (13.72 mmol) of 1,2-diaminoethane were placed in 20 ml of the same solvent (absolute EtOH) and slowly added. This mixture was heated to 50°C under stirring and nitrogen atmosphere for two hours. A precipitate obtained was filtered, washed with diethyl ether and then dried under reduced pressure to yield 4.26 g (58%) of the expected compound. Its melting point was found to be 156 °C and a suitable single-crystal was formed by slow evaporation from a solvent mixture EtOH/CH_2_Cl_2_ (8/2, *v*/*v*).

### Refinement

The remaining H atoms were localized on Fourier maps but introduced in calculated positions and treated as riding on their parent atoms (C and O) with C—H = 0.96 Å (methyl), 0.97 Å (methylene) or 0.93 Å (aromatic) and O—H = 0.82 Å with *U*_iso_(H) = 1.2*U*_eq_(C_aromatic_ and C_methylene_) or *U*_iso_(H) = 1.5*U*_eq_(C_methyl_ and O_hydroxy_).

### Crystal data

**Table:**

C_34_H_38_N_4_O_2_	*D*_x_ = 1.236 Mg m^−^^3^
*M**_r_* = 534.68	Mo *K*α radiation, λ = 0.71073 Å
Orthorhombic, *P**b**c**a*	Cell parameters from 3346 reflections
*a* = 7.460 (1) Å	θ = 1.0–26.4°
*b* = 12.350 (1) Å	µ = 0.08 mm^−^^1^
*c* = 31.176 (2) Å	*T* = 295 K
*V* = 2872.3 (5) Å^3^	Prism, yellow
*Z* = 4	0.15 × 0.08 × 0.04 mm
*F*(000) = 1144	

### Data collection

**Table:**

Nonius KappaCCD diffractometer	1698 reflections with *I* > 2σ(*I*)
Radiation source: Enraf–Nonius FR590	*R*_int_ = 0.023
Graphite monochromator	θ_max_ = 26.4°, θ_min_ = 3.3°
Detector resolution: 9 pixels mm^-1^	*h* = −9→9
CCD rotation images, thick slices scans	*k* = −15→15
5411 measured reflections	*l* = −38→38
2916 independent reflections	

### Refinement

**Table:**

Refinement on *F*^2^	Primary atom site location: structure-invariant direct methods
Least-squares matrix: full	Secondary atom site location: difference Fourier map
*R*[*F*^2^ > 2σ(*F*^2^)] = 0.063	Hydrogen site location: inferred from neighbouring sites
*wR*(*F*^2^) = 0.216	H-atom parameters constrained
*S* = 1.01	*w* = 1/[σ^2^(*F*_o_^2^) + (0.1401*P*)^2^] where *P* = (*F*_o_^2^ + 2*F*_c_^2^)/3
2916 reflections	(Δ/σ)_max_ < 0.001
185 parameters	Δρ_max_ = 0.30 e Å^−^^3^
0 restraints	Δρ_min_ = −0.15 e Å^−^^3^

### Special details

**Table:**

Geometry. All e.s.d.'s (except the e.s.d. in the dihedral angle between two l.s. planes) are estimated using the full covariance matrix. The cell e.s.d.'s are taken into account individually in the estimation of e.s.d.'s in distances, angles and torsion angles; correlations between e.s.d.'s in cell parameters are only used when they are defined by crystal symmetry. An approximate (isotropic) treatment of cell e.s.d.'s is used for estimating e.s.d.'s involving l.s. planes.
Refinement. Refinement of *F*^2^ against ALL reflections. The weighted *R*-factor *wR* and goodness of fit *S* are based on *F*^2^, conventional *R*-factors *R* are based on *F*, with *F* set to zero for negative *F*^2^. The threshold expression of *F*^2^ > σ(*F*^2^) is used only for calculating *R*-factors(gt) *etc*. and is not relevant to the choice of reflections for refinement. *R*-factors based on *F*^2^ are statistically about twice as large as those based on *F*, and *R*- factors based on ALL data will be even larger.

### Fractional atomic coordinates and isotropic or equivalent isotropic displacement parameters (Å^2^)

**Table:**

	*x*	*y*	*z*	*U*_iso_*/*U*_eq_	
C1	0.5460 (3)	0.46343 (16)	0.01583 (6)	0.0656 (6)	
H1A	0.469	0.4517	0.0405	0.079*	
H1B	0.5703	0.3938	0.0027	0.079*	
C2	0.8224 (3)	0.46551 (15)	0.05564 (6)	0.0596 (6)	
C3	0.7906 (3)	0.35392 (18)	0.07299 (8)	0.0807 (7)	
H3A	0.8621	0.3028	0.0573	0.121*	
H3B	0.6662	0.3357	0.07	0.121*	
H3C	0.8232	0.3519	0.1028	0.121*	
C4	0.9870 (2)	0.52384 (14)	0.06796 (6)	0.0570 (5)	
C5	1.1047 (3)	0.48234 (16)	0.09881 (6)	0.0629 (6)	
H5	1.0776	0.4159	0.1113	0.076*	
C6	1.2584 (3)	0.53460 (15)	0.11170 (7)	0.0667 (6)	
C7	1.2950 (3)	0.63504 (18)	0.09292 (7)	0.0742 (6)	
H7	1.3968	0.6731	0.1013	0.089*	
C8	1.1835 (3)	0.67844 (17)	0.06238 (7)	0.0724 (6)	
H8	1.2113	0.7452	0.0503	0.087*	
C9	1.0299 (3)	0.62421 (15)	0.04924 (6)	0.0610 (6)	
C10	1.3850 (3)	0.48353 (17)	0.14366 (8)	0.0820 (7)	
H10A	1.3408	0.4118	0.1506	0.098*	
H10B	1.5011	0.4749	0.1301	0.098*	
C11	1.2474 (3)	0.5513 (2)	0.20900 (8)	0.1033 (9)	
H11A	1.173	0.6082	0.1978	0.155*	
H11B	1.2779	0.5671	0.2383	0.155*	
H11C	1.1836	0.4839	0.2077	0.155*	
C12	1.5322 (3)	0.63015 (18)	0.18474 (6)	0.0679 (6)	
C13	1.6980 (3)	0.6251 (2)	0.16424 (7)	0.0792 (7)	
H13	1.7262	0.5643	0.148	0.095*	
C14	1.8194 (4)	0.7066 (3)	0.16743 (8)	0.0935 (8)	
H14	1.9291	0.7006	0.1534	0.112*	
C15	1.7821 (4)	0.7986 (2)	0.19121 (10)	0.1025 (9)	
H15	1.8654	0.8543	0.1934	0.123*	
C16	1.6205 (4)	0.8054 (2)	0.21135 (9)	0.0998 (9)	
H16	1.5936	0.8668	0.2274	0.12*	
C17	1.4965 (4)	0.7236 (2)	0.20856 (7)	0.0865 (8)	
H17	1.3873	0.7305	0.2227	0.104*	
N1	0.7139 (2)	0.51374 (13)	0.02949 (5)	0.0631 (5)	
N10	1.4097 (3)	0.54358 (15)	0.18363 (6)	0.0774 (6)	
O9	0.9279 (2)	0.66894 (11)	0.01863 (5)	0.0748 (5)	
H9	0.8365	0.6329	0.0155	0.112*	

### Atomic displacement parameters (Å^2^)

**Table:**

	*U*^11^	*U*^22^	*U*^33^	*U*^12^	*U*^13^	*U*^23^
C1	0.0617 (13)	0.0730 (13)	0.0621 (13)	−0.0004 (9)	−0.0044 (10)	−0.0032 (9)
C2	0.0630 (12)	0.0613 (12)	0.0544 (11)	0.0029 (9)	0.0016 (9)	−0.0035 (8)
C3	0.0839 (16)	0.0750 (14)	0.0831 (15)	−0.0086 (12)	−0.0155 (12)	0.0142 (11)
C4	0.0584 (12)	0.0608 (11)	0.0519 (11)	0.0026 (9)	0.0031 (9)	−0.0040 (8)
C5	0.0628 (13)	0.0633 (11)	0.0627 (12)	0.0037 (9)	−0.0031 (10)	0.0019 (9)
C6	0.0622 (13)	0.0719 (13)	0.0660 (13)	0.0061 (10)	−0.0056 (10)	−0.0045 (10)
C7	0.0658 (14)	0.0784 (14)	0.0785 (14)	−0.0068 (11)	−0.0063 (11)	−0.0007 (12)
C8	0.0716 (14)	0.0689 (13)	0.0766 (14)	−0.0077 (11)	0.0004 (12)	0.0065 (10)
C9	0.0636 (13)	0.0641 (12)	0.0553 (12)	0.0056 (10)	0.0011 (9)	−0.0013 (9)
C10	0.0747 (15)	0.0792 (13)	0.0919 (17)	0.0080 (12)	−0.0182 (13)	−0.0032 (13)
C11	0.0753 (17)	0.147 (3)	0.0877 (18)	−0.0085 (16)	0.0024 (15)	0.0101 (15)
C12	0.0622 (13)	0.0858 (14)	0.0557 (12)	0.0071 (11)	−0.0076 (10)	0.0053 (10)
C13	0.0705 (15)	0.0970 (16)	0.0702 (15)	0.0103 (12)	−0.0007 (11)	−0.0002 (12)
C14	0.0722 (16)	0.130 (2)	0.0779 (17)	−0.0072 (16)	−0.0056 (13)	0.0264 (16)
C15	0.111 (2)	0.107 (2)	0.0895 (19)	−0.0297 (18)	−0.0276 (17)	0.0273 (17)
C16	0.110 (2)	0.0918 (18)	0.097 (2)	0.0005 (16)	−0.0171 (17)	−0.0120 (14)
C17	0.0758 (16)	0.1022 (18)	0.0815 (18)	0.0120 (14)	−0.0016 (12)	−0.0065 (13)
N1	0.0610 (11)	0.0690 (10)	0.0595 (10)	0.0034 (8)	−0.0053 (8)	−0.0021 (8)
N10	0.0677 (12)	0.0937 (13)	0.0710 (12)	−0.0011 (10)	−0.0056 (9)	0.0019 (9)
O9	0.0808 (11)	0.0709 (9)	0.0726 (10)	0.0000 (8)	−0.0130 (8)	0.0109 (7)

### Geometric parameters (Å, º)

**Table:**

C1—N1	1.462 (3)	C10—N10	1.462 (3)
C1—C1^i^	1.503 (4)	C10—H10A	0.97
C1—H1A	0.97	C10—H10B	0.97
C1—H1B	0.97	C11—N10	1.449 (3)
C2—N1	1.294 (2)	C11—H11A	0.96
C2—C4	1.474 (3)	C11—H11B	0.96
C2—C3	1.499 (3)	C11—H11C	0.96
C3—H3A	0.96	C12—C13	1.393 (3)
C3—H3B	0.96	C12—C17	1.398 (3)
C3—H3C	0.96	C12—N10	1.407 (3)
C4—C5	1.400 (3)	C13—C14	1.358 (3)
C4—C9	1.407 (3)	C13—H13	0.93
C5—C6	1.376 (3)	C14—C15	1.384 (4)
C5—H5	0.93	C14—H14	0.93
C6—C7	1.399 (3)	C15—C16	1.362 (4)
C6—C10	1.511 (3)	C15—H15	0.93
C7—C8	1.373 (3)	C16—C17	1.372 (3)
C7—H7	0.93	C16—H16	0.93
C8—C9	1.389 (3)	C17—H17	0.93
C8—H8	0.93	O9—H9	0.82
C9—O9	1.340 (2)		
			
N1—C1—C1^i^	109.1 (2)	N10—C10—H10A	108.4
N1—C1—H1A	109.9	C6—C10—H10A	108.4
C1^i^—C1—H1A	109.9	N10—C10—H10B	108.4
N1—C1—H1B	109.9	C6—C10—H10B	108.4
C1^i^—C1—H1B	109.9	H10A—C10—H10B	107.5
H1A—C1—H1B	108.3	N10—C11—H11A	109.5
N1—C2—C4	117.40 (17)	N10—C11—H11B	109.5
N1—C2—C3	123.46 (19)	H11A—C11—H11B	109.5
C4—C2—C3	119.13 (18)	N10—C11—H11C	109.5
C2—C3—H3A	109.5	H11A—C11—H11C	109.5
C2—C3—H3B	109.5	H11B—C11—H11C	109.5
H3A—C3—H3B	109.5	C13—C12—C17	116.7 (2)
C2—C3—H3C	109.5	C13—C12—N10	122.1 (2)
H3A—C3—H3C	109.5	C17—C12—N10	121.1 (2)
H3B—C3—H3C	109.5	C14—C13—C12	121.7 (2)
C5—C4—C9	117.70 (18)	C14—C13—H13	119.2
C5—C4—C2	121.51 (17)	C12—C13—H13	119.2
C9—C4—C2	120.79 (17)	C13—C14—C15	120.9 (3)
C6—C5—C4	123.50 (19)	C13—C14—H14	119.6
C6—C5—H5	118.3	C15—C14—H14	119.6
C4—C5—H5	118.3	C16—C15—C14	118.4 (3)
C5—C6—C7	117.18 (19)	C16—C15—H15	120.8
C5—C6—C10	121.19 (18)	C14—C15—H15	120.8
C7—C6—C10	121.60 (19)	C15—C16—C17	121.5 (3)
C8—C7—C6	121.2 (2)	C15—C16—H16	119.3
C8—C7—H7	119.4	C17—C16—H16	119.3
C6—C7—H7	119.4	C16—C17—C12	120.9 (2)
C7—C8—C9	121.06 (19)	C16—C17—H17	119.6
C7—C8—H8	119.5	C12—C17—H17	119.6
C9—C8—H8	119.5	C2—N1—C1	121.60 (17)
O9—C9—C8	118.68 (17)	C12—N10—C11	118.62 (18)
O9—C9—C4	121.97 (18)	C12—N10—C10	119.24 (18)
C8—C9—C4	119.35 (19)	C11—N10—C10	113.17 (19)
N10—C10—C6	115.41 (18)	C9—O9—H9	109.5
			
N1—C2—C4—C5	174.50 (17)	C7—C6—C10—N10	63.5 (3)
C3—C2—C4—C5	−6.7 (3)	C17—C12—C13—C14	0.4 (3)
N1—C2—C4—C9	−4.9 (3)	N10—C12—C13—C14	−176.3 (2)
C3—C2—C4—C9	173.95 (18)	C12—C13—C14—C15	−0.3 (4)
C9—C4—C5—C6	0.3 (3)	C13—C14—C15—C16	0.0 (4)
C2—C4—C5—C6	−179.11 (17)	C14—C15—C16—C17	0.2 (4)
C4—C5—C6—C7	0.8 (3)	C15—C16—C17—C12	−0.1 (4)
C4—C5—C6—C10	−177.15 (18)	C13—C12—C17—C16	−0.2 (3)
C5—C6—C7—C8	−1.1 (3)	N10—C12—C17—C16	176.6 (2)
C10—C6—C7—C8	176.8 (2)	C4—C2—N1—C1	−179.07 (16)
C6—C7—C8—C9	0.3 (3)	C3—C2—N1—C1	2.2 (3)
C7—C8—C9—O9	−178.48 (18)	C1^i^—C1—N1—C2	−177.48 (19)
C7—C8—C9—C4	0.8 (3)	C13—C12—N10—C11	174.4 (2)
C5—C4—C9—O9	178.18 (17)	C17—C12—N10—C11	−2.2 (3)
C2—C4—C9—O9	−2.4 (3)	C13—C12—N10—C10	−40.6 (3)
C5—C4—C9—C8	−1.1 (3)	C17—C12—N10—C10	142.8 (2)
C2—C4—C9—C8	178.31 (17)	C6—C10—N10—C12	−82.4 (3)
C5—C6—C10—N10	−118.6 (2)	C6—C10—N10—C11	64.4 (2)

Symmetry code: (i) −*x*+1, −*y*+1, −*z*.

### Hydrogen-bond geometry (Å, º)

**Table:**

*D*—H···*A*	*D*—H	H···*A*	*D*···*A*	*D*—H···*A*
O9—H9···N1	0.82	1.79	2.517 (2)	147
